# Validity and reliability of a Fijian translation and adaptation of the Eating Disorder Examination Questionnaire

**DOI:** 10.1002/eat.20675

**Published:** 2009-03-23

**Authors:** Anne E Becker, Jennifer J Thomas, Asenaca Bainivualiku, Lauren Richards, Kesaia Navara, Andrea L Roberts, Stephen E Gilman, Ruth H Striegel-Moore, for the HEALTHY Fiji Study Group

**Affiliations:** 1 Department of Global Health and Social Medicine, Harvard Medical SchoolBoston, Massachusetts; 2 Eating Disorders Clinical and Research Program, Department of Psychiatry, Massachusetts General HospitalBoston, Massachusetts; 3 Klarman Eating Disorders Center, McLean HospitalBelmont, Massachusetts; 4 Department of Child & Youth Care, University of VictoriaVancouver, Canada; 5 Department of Psychology, Boston UniversityBoston, Massachusetts; 6 Narata VillageSigatoka, Fiji; 7 Department of Society, Human Development, and Health, Harvard School of Public HealthBoston, Massachusetts; 8 Department of Epidemiology, Harvard School of Public HealthBoston, Massachusetts; 9 Department of Psychology, Wesleyan UniversityMiddletown, Connecticut

**Keywords:** eating disorder, EDE-Q, validity, cross-cultural, Fiji

## Abstract

**Objective:**

Assessment of disordered eating has uncertain validity across culturally diverse populations. This study evaluated Eating Disorder Examination Questionnaire (EDE-Q) performance in an ethnic Fijian study population.

**Method:**

The EDE-Q was translated, adapted, and administered to school-going Fijian adolescent females (*N* = 523). A subsample (*n* = 81) completed it again within ∼1 week. We assessed feasibility, internal consistency, and test-retest reliability; evaluated construct validity through factor analysis and correlation with similar constructs; and examined the marginal utility of an additional question on traditional purgative use.

**Results:**

Internal consistency reliability was adequate for the global scale and subscales (Cronbach's alpha = 0.66–0.91); retest reliability was adequate for both the languages (range of ICCs, 0.50–0.79, and of kappas, 0.46–0.81, excluding purging items). Construct validity was supported by significant correlations with measures of similar constructs. Factor analysis confirms multiple dimensions of eating disorder symptoms but suggests possible culture-specific variation in this population. The majority of respondents endorsing traditional purgative use (58%) did not endorse conventional EDE-Q items assessing purging.

**Discussion:**

The EDE-Q is a valid measure of eating disorder pathology for ethnic Fijian adolescent females and measures a unitary underlying construct. © 2009 by Wiley Periodicals, Inc. Int J Eat Disord, 2010

## Introduction

Assessment of eating disorders across culturally diverse settings presents unique methodologic challenges. Foremost among these are the uncertain validity and public health significance of applying universal diagnostic criteria across socially diverse contexts-especially in the absence of indigenous nosologic eating disorder categories. DSM-IV diagnostic criteria may not capture locally meaningful expressions of distress or pathology. Moreover, distress and impairment—especially occupational and social impairment—related to symptoms is arguably context dependent.

Particular challenges to self-report assessment of eating disorders relate to operational definitions of binge eating,^1^ shape concerns, and even purging. Discrepancies between the intended and construed meanings of these constructs are potentially amplified when assessed across diverse settings for three key reasons. First, the translation of culturally unfamiliar attitudes and behaviors into the local, vernacular language may be limited by vocabulary or idiomatic expression. Second, the behaviors may be construed in a local context-specific way that does not relate to an eating disorder. For example, food preoccupation may relate to hunger and food deprivation rather than to weight concern.^2^ Third, the assessments may fail to capture local behavior or attitudes that are potentially pathological, but culturally unique, and therefore not assessed in standard questionnaires (i.e. rationales for food refusal that do not relate to weight concerns).^3^

Strategic considerations for assessment in non-Western settings center on minimizing classification bias by careful attention to translation and by maintaining flexibility in the identification and evaluation of symptoms. Specifically, avoiding skip-out questions to be inclusive of diverse patterns of presentation and adapting and augmenting questions to reflect indigenous practices, response styles, and local idioms may enhance flexibility. Dimensional assessment also allows flexibility in identifying clinically important patterns that could miss full-criteria definitions developed from knowledge based on European and North American populations.^4^ Finally, assessment must augment diagnostic capability of local practitioners by providing clinically useful data without placing additional strain on scarce health resources.

In this article, we report on the translation, adaptation, and psychometric evaluation of the Eating Disorder Examination self-report questionnaire (EDE-Q, version 5) in an adolescent female ethnic Fijian population in Fiji. The aims of this article are twofold: (1) to establish how the EDE-Q performs in this study population as a tool for identifying local mental health needs and risk correlates, and (2) to consider how findings may inform eating disorders assessment in diverse global settings. In other words, we wish to address a local public health need in Fiji as well as the dearth of empirical data evaluating psychometric properties of eating disorder assessment in non-Western settings.

We chose to study the EDE-Q because of its widespread use; development from the EDE interview—a semistructured investigator based assessment emerging as a gold standard for eating disorders assessment; assessment of the detailed information related to attitudes and behaviors; and generation of both dimensional and categorical DSM-IV diagnostic data.

## Method

### Study Site

The study was conducted among ethnic Fijians in Fiji. Ethnic Fijians are a small-scale indigenous population now undergoing rapid economic and social change. Traditionally, Fijian culture—drawing from its Polynesian and Melanesian origins—has supported robust appetite and body weight through a variety of cultural practices that maintain vigilance for appetite and weight loss and promote frequent feasting.^5^ Despite evidence that behavioral symptoms of disordered eating have increased concomitant with recent social change in Fiji,^6^,^7^ there is no Fijian indigenous nosologic category that corresponds to an eating disorder.

### Study Sample

The study population comprised all available ethnic Fijian adolescent females, ages 15-20, enrolled in Forms 3-6 in the 12 secondary schools registered in one administrative sector in the Fiji Ministry of Education as of October 2006.^8^ Participant eligibility was determined by administrative record review at each school and was amended by faculty report at the time of the survey. Eligibility criteria were confirmed by self-report at the time of the survey. A total of 523 eligible study participants were enrolled, representing a response rate of 71%.

### Self-Report Measures

#### Eating Disorder Examination-Questionnaire

The EDE-Q5.2 was the primary measure of disordered eating. This 28-item questionnaire assesses current frequency of bin-geing and purging behaviors and attitudinal symptoms (Restraint, Eating Concern, Weight Concern, and Shape Concern) over the past 28 days.^9^,^10^ To encompass the full range of purging behaviors that might be exhibited in our Fijian sample, we added an item to the EDE-Q assessing the presence of herbal purgative use that was identified through a previous focus group study conducted by the study investigators (A.E.B. and K.N.).

#### Global School-Based Student Health Survey

The GSHS^11^ is a survey adapted from the Centers for Disease Control's Youth Behavioral Risk Survey^12^ for the assessment of health risk behaviors among adolescents in globally diverse populations (adaptation/translation described in Becker et al., unpublished manuscript). Included were five yes/no items assessing weight loss efforts by dieting, exercise, fasting, and purging in the past 30 days as well as items assessing weight gain attempts and hunger because of inadequate food in the household. Reliability for this assessment is reported elsewhere; the mean kappa for retest of items relating to dietary behaviors was 0.38 (Becker et al., unpublished manuscript).

#### Body Figure Scale

The BFS is a two-item scale asking respondents to select one of nine cartoon adult female body figures (ranging from thin to obese) that corresponds to their perceived actual body shape and ideal body shape.^13^ We calculated a score for each respondent to reflect the perceived discrepancy between ideal and actual body figure size. A score > 0 indicated that the participant's current shape was larger than her ideal, a score of 0 indicated no discrepancy, and a score \ 0 indicated that her body is thinner than her ideal.

#### Body Esteem Scale for Adults and Adolescents, Weight Subscale

We measured body weight satisfaction with the eight-item self-report BESAA Weight Subscale, on which higher scores indicate greater satisfaction.^14^

#### Fijian Body Shape Concern and Dissatisfaction Questionnaire

The FBSQ is a six-item measure assessing body dissatisfaction; of these, five items are Likert style with four response options and one forces a yes/no response. A previous version of this questionnaire has shown acceptable internal consistency reliability in a similar study population.^15^ Higher scores reflect greater shape and weight concerns.

#### Questions on Tradition and Change

Two items developed previously for a similar study population were used to assess the frequency of overeating at traditional feasts and the extent to which this bothered participants as three to four ordinal response options. Higher scores reflect overeating at feasts more frequently and feeling more bothered by this behavior, respectively.

#### Translation of the Study Instruments

Before translation, the EDE-Q was reviewed for items that might present conceptual challenges based on input from local informants and previous ethnographic work by the first author (A.E.B.). The adapted EDE-Q was translated into the local vernacular Fijian language, edited for syntax, and then back-translated by a bilingual Fiji language scholar. Original and back-translated versions were reviewed, compared, and adjusted for equivalence by consensus among the study team and consultant linguist, with particular attention to achieving the most idiomatic means possible for conveying the intended clinical meanings of “binge,” “purge,” and “calories.” Additional edits were made for clarity, and the final Fijian language translation was reviewed by a native speaker (K.N.) for grammatical and semantic accuracy. The GSHS, BFS, FBSQ, QTC, and BESAA weight subscale were translated into the Fijian language following a similar protocol.

### Study Procedures

Respondents and parents were informed that participants whose responses signaled potentially serious concerns about their health—including a potential eating disorder—would be invited for an interview and put in touch with a responsible adult—but not a parent—at their schools for further assistance. After parental informed consent and participant assent were obtained, study participants were oriented to the self-report assessment, which was one of numerous other assessments in a booklet they were asked to complete. Guided by their experience with difficulties in translation, the study team highlighted and defined potentially unfamiliar terms as part of the orientation process and encouraged respondents to request additional ad hoc clarification as needed during the proctored administration of the survey. Respondents were offered a choice of either an English or Fijian language version of the assessment battery. Psychometric evaluation of assessments was a component of a study protocol that was approved by the Fiji National Research Ethical Review Committee (FN-RERC), the Partners Healthcare Human Subjects Committee, and the Harvard Medical School Committee on Human Studies.

#### Missing Data

After returning the survey, respondents were given an opportunity to respond to or clarify their preferred answer in the case of missing or double-entered responses identified. Per EDE-Q scoring instructions, subscale scores were calculated as long as participants omitted responses to no more than two Restraint items, two Eating Concern items, two Weight Concern items, or four Shape Concern items. Global scores were calculated as long as participants completed at least two subscales.

#### Retesting

Respondents at Time 1 in 3 of the 12 schools—purposively selected to represent each of three geographic regions with associated linguistic diversity— were invited to respond to the battery within ∼1 week (i.e., at Time 2) under similar conditions and in the same language as they had selected at Time 1. Respondents absent on the retesting day were excluded. All eligible respondents participated in retesting.

### Statistical Analyses

We evaluated the performance of the EDE-Q in our ethnic Fijian sample with respect to feasibility, reliability, and validity. We assessed the feasibility of EDE-Q administration in our sample by calculating the percentage of questionnaires participants returned with sufficient completed data for scoring. We assessed reliability by examining internal consistency reliability in the entire sample as well as retest reliability in a subsample (*n* = 81) who repeated the EDE-Q at Time 2. Internal consistency reliability was estimated by calculating Cronbach's alpha coefficients for each of the EDE-Q subscales as well as the global score. Test-retest reliability was estimated by calculating intraclass correlation coefficients for continuous variables and Cohen's kappa for categorical variables. Internal consistency and test-retest reliability were calculated separately for the English language and Fijian language subsamples. We assessed construct validity by examining correlations of EDE-Q subscale scores, global scores, and individual binge-eating/purging items with measures of conceptually overlapping constructs. These included items referencing similar content on the GSHS, QTC, BFS, FBSQ, and BESAA. To test our hypothesis that adding a question about traditional herbal purgative use could enhance detection of symptomatic behavior, we calculated the percentage of participants who would have been classified as nonpurging via the standard EDE-Q purging items assessing vomiting and laxative use alone. Finally, we examined the factor structure of the EDE-Q in an exploratory factor analysis of all the Likert-style items of the EDE-Q that comprise the global score. We applied oblique promax rotation to account for the anticipated intercorrelation among factors, and fitted a four-factor model in an attempt to replicate the predicted Restraint, Eating Concern, Weight Concern, and Shape Concern subscales.

## Results

The study sample comprised ethnic Fijian adolescent females enrolled in secondary schools within one administrative division on the main island of Fiji. The sample characteristics are summarized in Table 1.

**Table 1:** Demographic characteristics of 523 school-going Fijian adolescent females

	Mean (SD)
Age	16.67 (1.09) years
Body mass index	23.97 (3.35) kg/m^2^
	Frequency (percent)
Community population density	
Urban	261 (49.90%)
Rural	262(50.10%)
School level	
Form 3	42 (8.03%)
Form 4	161 (30.77%)
Form 5	160(30.60%)
Form 6	160(30.60%)
Language of questionnaire	
English	147(28.11%)
Fijian (local dialect)	376(71.89%)

### Feasibility

A large percentage (*n* = 432, 82.60%) of the EDE-Qs was returned with missing data. However, excluding the items assessing self-reported height and weight, 478 (91.40%) of the questionnaires were completed in their entirety. For all but one participant, the data returned allowed the calculation of global and subscale scores.

### Internal Consistency Reliability

Cronbach's alphas for both English and Fijian language versions of the EDE-Q showed adequate to excellent internal consistency reliability ranging from 0.65 to 0.91 for the English language version and from 0.66 to 0.81 for the Fijian language version (Table 2).

**Table 2:** Internal consistency and test-retest reliability for continuous and categorical items for participants who completed English or Fijian language versions of the EDE-Q

	English		Fijian	
	Continuous Items			
	Time 1 Cronbach's α (*n* = 140–146)^a^	Test-Retest ICC (*n* = 21)	Time 1 Cronbach's α (*n* = 360–374)^a^	Test-Retest ICC (*n* = 60)
EDE-Q Global	0.91	0.79	0.81	0.70
EDE-Q Restraint	0.70	0.75	0.72	0.60
EDE-Q Eating Concern	0.65	0.55	0.66	0.50
EDE-Q Shape Concern	0.84	0.70	0.79	0.63
EDE-Q Weight Concern	0.70	0.78	0.66	0.56
	Categorical Items			
	*k*	% Agreement	*k*	% Agreement

Any purging	0.81	90.5	0.62	83.0
Vomiting	0.39	76.2	0.66	91.3
Laxative misuse	0.48	81.0	0.13	86.0
Herbal purgative use	0.51	76.2	0.63	86.4
Driven exercise	0.53	76.2	0.46	73.7
Fasting	0.69	85.7	0.61	90.0
Binge eating	0.55	80.9	0.60	83.3

Notes: EDE-Q, Eating Disorder Examination Questionnaire; “Any purging” is defined here as an affirmative response to at least one item probing vomiting, laxative misuse, or herbal purgative use.

a Sample sizes vary per subscale because not all participants answered all EDE-Q items.

### Test-Retest Reliability

Eighty-one respondents completed a second EDE-Q at Time 2. Twenty-one students completed a retest of the English language version (14% of English language test-takers) and 60 completed a retest of the Fijian language version (16% of Fijian language test-takers). Intraclass correlation coefficients indicating the test-retest reliability between each of the four subscales and the global score are displayed in Table 2. Kappa coefficients indicating the test-retest reliability of the items assessing vomiting, laxative use, herbal purgative use, any purging, driven exercise, fasting, and binge eating ranged from fair to substantial (0.39 to 0.81; percent agreement 73 to 91%). However, test-retest reliability of laxative use was poor in the Fijian language version (*k* = 0.13; 86.0% agreement; Table 2).

### Construct Validity

As hypothesized, EDE-Q subscale scores and individual binge-eating/purging items exhibited positive correlations with items assessing similar constructs on the GSHS, BFS, FBSQ, and QTC. The majority of these correlations were medium to large and all were statistically significant. Medium to large negative correlations with the BESAA weight subscale also supported construct validity of the weight and shape concern subscales. The EDE-Q global score also was positively and significantly correlated with the presence of vomiting, laxative use, herbal purgative use, driven exercise, and binge eating on the EDE-Q. The consistently significant correlations between EDE-Q measures and similar constructs provided support for the construct validity of the EDE-Q in our sample (Table 3). As expected, the four EDE-Q subscales also exhibited moderate to high intercorrelations with one another (ranging from 0.42 to 0.83), and all of these were statistically significant.

**Table 3:** Correlations between EDE-Q and constructs measuring similar attitudes and behaviors

EDE-Q Item or Scale	Construct	Pearson Correlation (*r*)
EDE-Q Restraint	GSHS fasting	.27**
	GSHS dieting	.28**
	GSHS current weight loss attempt	.26**
EDE-Q Eating concern	QTC overeating at feasts	.23**
	QTC bothered by overeating at feasts	.18**
	EDE-Q any binge eating	.26**
	EDE-Q loss of control during overeating episodes	.28**
EDE-Q Shape concern	Discrepancy between ideal and actual shape on BFS	.38**
	FBSQ	.51**
	BESAA—Weight subscale	−.48**
EDE-Q Weight concern	Discrepancy between ideal and actual shape on BFS	.36**
	FBSQ	.53**
	BESAA—Weight subscale	−.43**
EDE-Q Global	GSHS trying to lose weight	.38**
	EDE-Q vomiting	.19**
	EDE-Q laxatives	.15**
	EDE-Q herbal purgative use	.20**
	EDE-Q driven exercise	.34**
	EDE-Q binge eating	.24**
EDE-Q purging by vomiting or laxatives	GSHS purging by vomiting or laxatives	.28**
EDE-Q any purging	GSHS purging by vomiting or laxatives	.21**
EDE-Q driven exercise	GSHS exercise	.42**
EDE-Q fasting (8 h)	GSHS fasting (24 h)	.31**
EDE-Q dieting	GSHS eating less food, fewer calories, or low fat food	.29**
	GSHS trying to lose weight	.24**
EDE-Q binge eating	QTC overeating at feasts	.11*

Notes: EDE-Q, Eating Disorder Examination Questionnaire; GSHS, Global School-Based Student Health Survey; QTC, Questions about traditions and change; BFS, Body Figure Scale; FBSQ, Fijian Body Shape Concern and Dissatisfaction Questionnaire; BESAA, Body Esteem Scale for Adults and Adolescents; EDE-Q any purging = vomiting, laxative misuse, and/or herbal purgative use.

* *p*<.05;

** *p*<.001.

Approximately one third (35%, *n* =183) of participants who answered all items probing purging on the EDE-Q self-reported traditional herbal purgative use. Of these, only 42% (*n* = 76,) screened positive for conventional EDE-Q items assessing purging, including 17% (*n* = 13) who self-reported laxative misuse, 45%, (*n* = 34) who reported vomiting, and 38% (*n* = 29) who reported both vomiting and laxative misuse. Therefore, of those who self-reported herbal purgative use, 58% (*n* = 107) would have been classified as nonpurging on the standard version of the EDE-Q by their negative response to vomiting or laxative misuse.

### Factor Structure from Exploratory Factor Analysis

**FIGURE 1:**
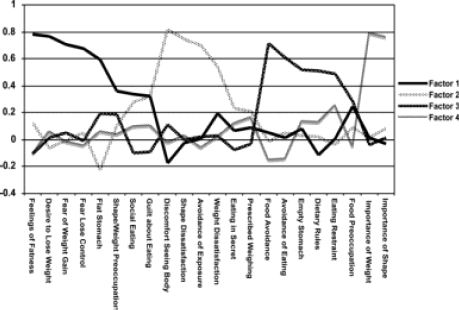
Loadings on each of the four factors derived from an exploratory factor analysis of the EDE-Q in an ethnic Fijian population

Principal axis factoring requesting a four-factor solution of the items assessed by Likert scales (but not the symptom frequency items) produced four factors accounting for 42.46% of the variance. Factor loadings on each of the four factors are displayed in Figure 1, with four separate lines indicating the pattern matrix factor loading of each item on that particular factor. Intercorrelations among factors ranged from medium to large, supporting the overarching coherence of the global scale (Table 4). Of the individual factors, the first (represented by the first peak of elevated factor loadings in Fig. 1) explained 29.82% of the variance and contained items from the Shape Concern, Weight Concern, and Eating Concern subscales that focused on fears of weight gain and the desire to change body shape. The second (peak 2 in Fig. 1) accounted for 5.92% of the variance, and featured items pertaining to dissatisfaction, shame, and discomfort with the body. The third factor (peak 3, 3.75% of the variance) resembled the Restraint sub-scale and also included one item from the Eating Concern scale assessing preoccupation with food, eating, or calories. The last factor contained the two items assessing the use of shape and weight information in self-evaluation (peak 4, 2.96% of the variance). Three items (“How much would it have upset you if you had been asked to weigh yourself once a week?”; “Has thinking about food, eating, or calories made it very difficult for you to concentrate on things you are interested in?”; and “On how many days have you eaten in secret?”) did not load highly on any factor.

**Table 4:** Inter-correlations among factors in EDE-Q factor analysis

	Factor 1	Factor 2	Factor 3	Factor 4
Factor 1	1.00	0.57**	0.58**	0.62**
Factor 2		1.00	0.38**	0.54**
Factor 3			1.00	0.49**
Factor 4				1.00

Notes: EDE-Q, Eating Disorder Examination Questionnaire.

*p<.05; ** *p*<.001.

## Discussion

This adaptation and translation of the EDE-Q demonstrated adequate internal consistency and test-retest reliability as well as construct validity in our ethnic Fijian sample, providing support for its use as a global measure of eating pathology in this population. High Cronbach's alpha coefficients for the global, Restraint, and Shape Concern scales are comparable with those observed in Western samples.^16^,^18^ Internal consistency was somewhat lower for the Eating Concern and Weight Concern sub-scales, which may reflect less cultural relevance of the three items with low loadings in the factor analysis. In keeping with previous investigations of EDE-Q test-retest reliability in Western samples,^17^,^19^ we observed greater temporal stability for continuous EDE-Q subscales measuring attitudinal components of eating pathology than for individual items measuring binge eating and purging behaviors. Because the EDE-Q frames questions in terms of the past 28 days, the lower reliability of behavioral items may reflect not just measurement error, but also true changes in the prevalence of disordered eating behaviors over the 1-week test interval.^19^

The fact that the EDE-Q global scale, subscale scores, and individual behavioral items correlated positively and significantly with all measures of similar constructs (such as body satisfaction, discrepancy between perceived ideal and self body shape, and individual items on the GSHS) support the construct validity of the EDE-Q in our sample. However, the substantial proportion of participants (58%) who would have been classified as nonpurg-ing had we not added an item on herbal purgative use warrants caution about the capacity for standard EDE-Q behavioral items to adequately encompass and identify the full range of eating pathology in non-Western populations. Because this item did not allow us to distinguish medicinal and weight management goals motivating the use of herbal purgatives nor to evaluate whether the behavior was consistent with misuse according to local social norms, these self-report data are insufficient to confirm misclassification of cases as nonpurg-ing. However, the high frequency of affirmative responses underscores the importance of examining local weight control strategies in relation to social norms in evaluating prevalence of eating disorders across diverse cultural settings.

The results of our exploratory factor analysis are also consistent with the interpretation of global EDE-Q scores as a unitary construct in our study population. Although we identified some evidence of underlying multidimensionality in the form of four factors, all were moderately to highly correlated, and we also obtained a high Cronbach's alpha for the EDE-Q global score. Consistent with the findings of previous factor analyses of the EDE and EDE-Q in Western samples,^16^,^20^–^22^ we found some support for the construct validity of a distinct Restraint subscale. However, as in previous studies^16^,^20^–^22^ our empirically derived factor structure departed somewhat from the clinically hypothesized four-subscale solution. The finding that the two self-evaluation items composed their own factor (rather than loading separately onto Shape and Weight Concerns, respectively) replicates the findings of two prior factor analyses^16^,^20^ and supports that theoretical possibility that *investment in* versus *satisfaction with* appearance represent two distinct facets of body image. Of course, this putative factor should be interpreted cautiously because of its small number of items and the small proportion of unique variance it explained. The items that exhibited the lowest loadings in our factor analysis may provide interesting insights into the phenomenology of eating pathology in the study population. One of these, reaction to prescribed weighing, exhibited similarly low loadings in two previous studies^16^,^20^ and may have diminished psychometric utility in populations exhibiting wide economic and cultural diversity. Because 76% of participants in the current study did not know their weight at the time of testing, reaction to prescribed weighing may not bear the same affective significance as in Western populations. Analogously, the item probing eating in secret may be less relevant to of eating pathology in a population where communal feasting is commonplace and eating secretly is stigmatized. Finally, preoccupation with food, eating, or calories may have been difficult for respondents unfamiliar with the concept of “calories” to understand. It may also have tapped food preoccupation related to hunger, which was prevalent in the study population.

Our results should be interpreted in the context of the following limitations. First, we selected a short between-test interval to ensure that the first and second test administrations would measure overlapping four-week timeframes. However, it is possible that participants may have recalled and subsequently restated their original responses at Time 2 (thus inflating retest reliability) or that viewing test items at baseline may have heightened awareness of shape and weight concerns at Time 2 (thus reducing retest reliability). Finally, it is possible that interviews conducted with some respondents at Time 2 may have influenced their Time 2 self-report responses. With regard to construct validity, we did not ask participants about diuretic use, and inquiring about alternative methods of purging might have improved its detection by standard EDE-Q items and thus reduced the marginal utility of asking about herbal purgative use. Alternatively, the item probing herbal purgative may underestimate the true prevalence of the behavior, because it did not specify intentional induction of vomiting. Another key limitation of this item is that it did not distinguish socially normative traditional purgative use from misuse that is consistent with purging associated with an eating disorder, and future studies should further evaluate traditional purgative use and its relation to disordered eating. Finally, our conclusions about construct validity rest on the assumption that the measures of similar constructs were valid in this study population. With the exception of the low reliability of some GSHS items, our data (available by request from the corresponding author) support adequate reliability for the other measures used to assess construct validity.

Our study supports that the modified Fijian version of the EDE-Q—in both its English and Fijian language versions—is a valid measure of eating disorder psychopathology in our ethnic Fijian school-going adolescent female population. Our data also support the use of the EDE-Q global summary score as a unitary and dimensional construct in indicating eating disorder pathology for this population. Our results also suggest that including a culture-specific item that relates to local weight management behaviors may enhance detection of eating disorder symptoms. Methodologic challenges in cross-cultural assessment of mental illness are not unique to measurement of eating disorders. However, we suggest that interpretation of behaviors as pathological must be made in consideration of the context of local social norms.

Eating disorders have global reach and populations undergoing economic and social transition related to urbanization, globalization, and migration may be at increased risk. Culturally sensitive and locally valid assessments for eating disorders are therefore essential to addressing local health concerns in the global context of escalating need. In addition, comparative cultural data on prevalence and phenomenology of disordered eating have relevance to mapping causal pathways by identifying contributions of social environment to risk and clinical presentation. This cross-cultural comparison will assist in characterizing potentially diverse presentations of illness, reduce mis-classification of culturally unique presentations, and open channels for identifying culture-specific and culturally sensitive prevention and intervention strategies.
